# *Diutina cutanea* sp. nov.—phylogenomic-based analysis of a novel clinically relevant ascomycetous yeast

**DOI:** 10.1093/mmy/myag029

**Published:** 2026-04-14

**Authors:** Sander Boden, Annemarie Zandijk, Shawn R Lockhart, Tjomme van der Bruggen, Bert Gerrits van den Ende, Marizeth Groenewald, Ferry Hagen

**Affiliations:** Research Group Analysis Techniques in the Life Sciences, Centre of Expertise Perspective in Health, Avans University of Applied Science, 4818AJ Breda, The Netherlands; Westerdijk Fungal Biodiversity Institute, 3584CT Utrecht, The Netherlands; Mycotic Diseases Branch, Centers for Disease Control & Prevention, 30341-3717 Atlanta, Georgia, USA; Department of Medical Microbiology, University Medical Center Utrecht, 3584CX Utrecht, The Netherlands; Westerdijk Fungal Biodiversity Institute, 3584CT Utrecht, The Netherlands; Westerdijk Fungal Biodiversity Institute, 3584CT Utrecht, The Netherlands; Westerdijk Fungal Biodiversity Institute, 3584CT Utrecht, The Netherlands; Department of Medical Microbiology, University Medical Center Utrecht, 3584CX Utrecht, The Netherlands

**Keywords:** chromogenic medium, cutaneous infection, nanopore sequencing, Saccharomycotina, MALDI-TOF MS

## Abstract

Ascomycetous yeasts of the genus *Diutina* are rarely encountered in clinical routine diagnostics, but known to harbor species with decreased susceptibility to azole antifungals. This study describes a clinical strain representing a novel member of the genus *Diutina*, which we named *Diutina cutanea*. The clinical strain, isolated from a human foot wound, was molecularly identified by ITS/D1D2 ribosomal DNA sequencing. Extensive phenotypic characterization was done by culturing, microscopy, sporulation assays, assimilation tests, and antifungal susceptibility testing. Nanopore sequencing was performed for *de novo* genome assembly and comparative genomics. ITS/D1D2 ribosomal DNA sequencing resulted in a low identity score compared to other *Diutina* species. Morphological characterization showed round to oval cells of 2–3 × 3–8 µm in size, multilateral budding, and formation of pseudohyphae were observed. Colonies were white, glossy, smooth, butyrous, and had an entire margin. Growth on CHROMagar Candida Plus allowed phenotypic distinction between *D. cutanea* and related clinically relevant species. Antifungal susceptibility showed an elevated fluconazole MIC of 4 µg/ml. Genome sequencing placed *D. cutanea* distantly but basal to the non-pathogenic relatives *D. scorzettiae, D. ranongensis*, and *D. siamensis*. ANI analysis resulted in a pairwise similarity of 0.83–0.86 between *D. cutanea* and its relatives. In conclusion, phylogenomic and ANI analyses showed *D. cutanea* to be a unique taxon within the genus *Diutina. Diutina cutanea* can be reliably phenotypically discerned from other clinically relevant *Diutina* species by culturing on CHROMagar Candida Plus.

## Introduction

A wide spectrum of ascomycetous yeast species within the subphylum Saccharomycotina are known to be (opportunistic) human fungal pathogens. The major species causing infections are *Candida albicans, Candida parapsilosis, Candida tropicalis, Nakaseomyces glabratus*, and *Pichia kudriavzevii*, followed by more than 30 relatively rare species that are dispersed over the phylogenetic tree of Saccharomycotina yeasts.^1^ One of these rarely encountered genera in clinical routine diagnostics is the genus *Diutina*, known to harbor species that have decreased susceptibility or resistance to azole antifungals.^1,2^ The genus *Diutina* was established a decade ago to accommodate a group of closely related *Candida* species, including *Diutina catenulata, D. mesorugosa, D. neorugosa, D. pseudorugosa, D. ranongensis, D. rugosa, D. scorzettiae*, and *D. siamensis*.^3^ A subsequent study on clinically relevant *Diutina* species reported that *D. mesorugosa* could not be reliably distinguished from *D. rugosa*, and hence it was suggested that they are synonyms of the same species.^4^*Candida pararugosa* was once known as a close relative of *D. rugosa*, but with the advent of molecular techniques, it was found to be genetically distinct, and it has been placed in the genus *Wickerhamiella* as *Wickerhamiella pararugosa*.^5^ Next to sequence-based approaches, the use of matrix-assisted laser desorption/ionization time-of-flight mass spectrometry (MALDI-TOF MS) has become a rapid and cost-effective tool to identify rare yeasts, like *Diutina*. However, low identity scores have been reported due to the low number of strains per species for which a reference spectrum is available in the database that comes with this commercial identification platform. Hence, enrichment of in-house databases is needed to more reliably identify *Diutina* and other rare yeasts.^6^ Over the past few years, fluconazole-resistant *D. catenulata* strains have increasingly been reported from clinical specimens and environments, including a cluster of infections in a Brazilian tertiary hospital.^7–10^ In the present study, we describe a clinical strain that represents a novel member of the genus *Diutina*.

## Material and methods

### Strain collection and molecular identification

The clinical strain B17938 was obtained from a human foot wound at the fifth toe, collected in May 2019 in Minnesota, USA, and sent to the Mycotic Diseases Branch of the Centers for Diseases Control and Prevention (CDC), Atlanta, GA, USA. No other clinically relevant information could be obtained. At the CDC, the strain was identified as *Candida* species based on ITS ribosomal DNA sequencing. Repeating this molecular identification, as well as additional phylogenetic and phenotypic investigations, was done at the Westerdijk Fungal Biodiversity Institute, Utrecht, The Netherlands. Here, the strain was deposited in the CBS Culture Collection as CBS 19460^T^ (= 2MG-A1704-35 = B17938). The strain was maintained on malt-extract agar (MEA; Oxoid, Basingstoke, UK) for future phenotypic and genomic analyses.

Genomic DNA was extracted using a rapid cetyltrimethylammonium bromide-based method as previously described.^11^ The internal transcribed spacer (ITS) and the partial 26S large subunit of the ribosomal DNA (D1/D2) of CBS 19460^T^ were amplified as previously described.^12^ The obtained raw sequence reads were manually checked and assembled into a single contig sequence using Lasergene SeqMan v17 (DNASTAR, Madison, WI, USA).

### Phenotypic characterization

The colony morphology of strain CBS 19460^T^ was determined using plate cultures grown on 2% glucose, 0.5% yeast extract, 1% peptone, and 1.5% agar (GYPA) for 7 days at 25°C. Microscopic pictures were taken using an Axioskop 2 plus (Carl Zeiss, Jena, Germany) microscope fitted with a Nikon DS-Ri2 microscope camera (Nikon Instruments, Melville, NY, USA). Cells grown on GYPA at 25°C for 7 days, and pseudohyphae grown using slide cultures for 7 days at 25°C on GYPA were photographed.^13^ Ascosporulation was tested on V8-juice agar (V8), yeast extract–malt extract agar, potato dextrose agar, cornmeal agar, and Difco malt extract agar, all incubated at 25°C for up to 1 month, while fermentation and nitrogen assimilation were assessed using the Durham tube and the auxanographic methods, respectively, following previously established protocols.^13^ Assimilation of carbohydrates was performed using API32C strips (bioMérieux, Marcy-l’Étoile, France) and incubated at 25°C for up to 14 days.

Furthermore, the strain CBS 19460^T^ was inoculated onto CHROMagar Candida Plus (CHROMagar, Paris, France) and incubated for 48 h at 35°C, together with the type strains of *D. catenulata* CBS 565^T^, *D. pseudorugosa* CBS 10433^T^, and *D. rugosa* CBS 613^T^.

Antifungal susceptibility testing was performed using the SensiTitre YeastOne YO10 AST plate according to the instructions provided by the manufacturer (Thermo Fisher Scientific, Waltham, MA, USA). The YO10 AST plates include the following antifungal compounds and their respective ranges: amphotericin B (0.12–8 µg/mg), 5-fluorocytosine (0.06–64 µg/ml), anidulafungin (0.015–8 µg/ml), caspofungin and micafungin (0.008–8 µg/ml), fluconazole (0.12–256 µg/ml), itraconazole (0.15–16 µg/ml), and posaconazole and voriconazole (0.008–8 µg/ml).

### Genome sequencing

Genomic DNA was extracted from a 72-h-old culture onto MEA using a previously described protocol that is based on purification by cetyltrimethylammonium bromide solution.^14^ Quality and quantity of genomic DNA were assessed by fluorometric quantification using the Qubit High Sensitivity kit (Thermo Fisher Scientific), 1% agarose gel electrophoresis with a 200 bp–10 kbp Smart Ladder (Eurogentec, Seraing, Belgium), and TapeStation Genomic DNA ScreenTape (Agilent, Santa Clara, CA, USA). Subsequently, nanopore long-read sequencing was performed for which 1000 ng of genomic DNA was used as input for the library preparation with the native ligation barcoding kit SQK-NBD114.24 followed by sequencing onto the GridION platform using a MinION R10.4.1 flow cell (all Oxford Nanopore Technologies (ONT), Oxford, United Kingdom). Raw data were basecalled with Dorado v7.6.7 in super accuracy mode using the basecalling model dna_r10.4.1_e8.2_400bps_sup@v5.0.0 (ONT). Basecalled data were collected into a single FASTQ file and filtered using chopper v0.8.0 for length (≥1100 bp) and quality (*Q* ≥ 10) after which 50 bp of the 5′- and 3′-ends were arbitrarily removed.^15^

### Genome analysis

The genome of strain CBS 19460^T^ was *de novo* assembled using Flye v2.9.5-b1801, and the obtained assembly was manually curated to check for barcode-leakage-related fragments from other samples within the same multiplex genome sequencing run, as well as to check the circular mitochondrial genome for an introduced overlapping sequence.^16^ To analyze the completeness of the obtained genome, a BUSCO analysis was conducted using compleasm v0.2.7 using the Ascomycota and Sacharomycetes lineages.^17^ A prediction of the number of genes was performed using the web-based version of Helixer v0.3.4 for which the fungi_v0.3_a_0100 model was used.^18^

### Phylogenomic analysis

The *de novo* assembled genome of CBS 19460^T^ was compared to the reference genomes of all accepted *Diutina* species present in NCBI Genome, the used assembly version and BioProject accession number are indicated in the phylogenetic tree. As outgroup, we used the reference genome of *Clavispora lusitaniae* strain FDAARGOS_655 that consists of eight contigs and had a genome size of 12.1 Mbp. Genome assemblies were first analyzed for completeness using compleasm v0.2.7 with the *Debaryomycetacae* database, all were found to be >80% complete.^17^ Thereafter, Helixer v0.3.64 was used with the fungi_v0.3_a_0100 model for gene/protein prediction prior running OrthoFinder v3.1.0 with default settings.^18,19^ The phylogenetic tree was subsequently enriched with metadata using Adobe Illustrator 2025 (Adobe Systems, San José, CA, USA).

### Average nucleotide identity analysis

To calculate the average nucleotide identity (ANI) between each possible pair of genomes within the *Diutina* genome dataset, the tool pyani v0.3.0-alpha was applied, which calculates a tree based on the obtained ANI-value matrix.^20^

#### MALDI-TOF MS reference spectrum

Strain CBS 19460^T^ was subcultured on MEA medium and incubated for 48 h at 35°C, and a mass spectrum profile (MSP) was created using the Bruker MALDI Biotyper Smart platform following the protocol provided by the manufacturer (Bruker Daltonics, Bremen, Germany).

## Results

### Molecular identification

The obtained ITS and D1/D2 sequences of strain CBS 19460^T^ were analyzed with the NCBI GenBank BLAST tool and resulted, despite 100% query coverage, in low identity scores of ≤89% with *Diutina* species, which is indicative that strain CBS 19460^T^ is potentially a novel species, formally described as *D. cutanea* in the taxonomic section. The sequence has been deposited in NCBI GenBank with accession number PX583673.

### Phenotypic characterization

**Figure 1.**
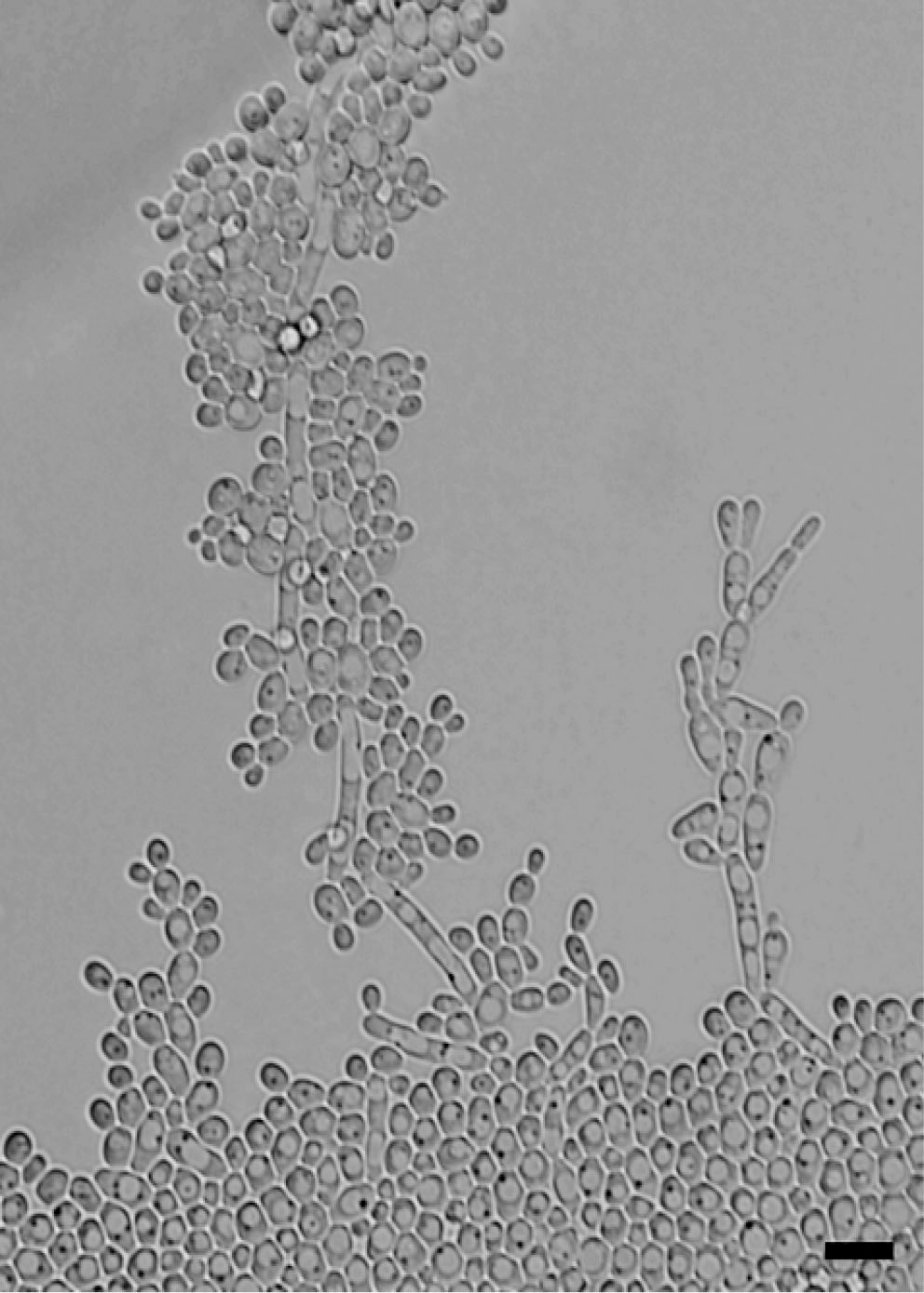
*Diutina cutanea* sp. nov. CBS 19460^T^. Slide cultures, showing vegetative cells and the formation of pseudohyphae after 7 days at 25°C on GYPA. Bar represents 10 µm.

The physiological characters, including the carbon and nitrogen assimilation, fermentation, and temperature ranges, are listed in the taxonomic description. Slide cultures showed pseudohyphae formation at 25°C on GYPA after 7 days of incubation (Fig. 1).

**Figure 2.**
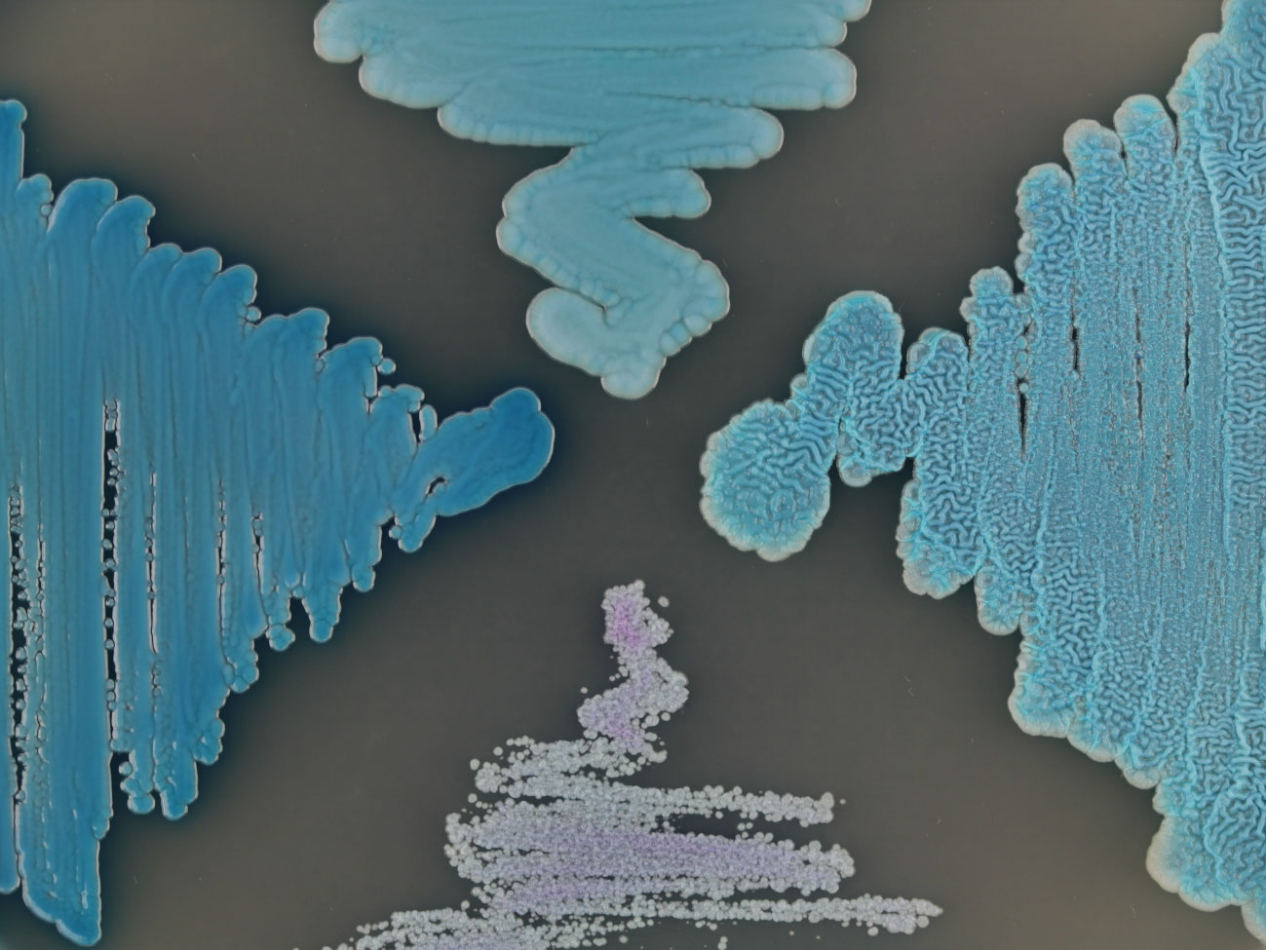
Clinically relevant *Diutina* species on CHROMagar Candida Plus medium incubated for 48 h at 35°C. Top: *Diutina rugosa* CBS 613^T^ bright blue smooth colonies; right: *Diutina pseudorugosa* CBS 10433^T^ bright blue wrinkled colonies; bottom: *Diutina catenulata* CBS 565^T^ pale purple-colored smooth colonies; left: *Diutina cutanea* CBS 19460^T^ dark blue smooth colonies. None of these *Diutina* species forms a halo in the medium.

On CHROMagar Candida Plus, the colonies of *D. rugosa* appear to be bright blue and smooth, similarly for *D. pseudorugosa*, although these colonies have a wrinkled surface, *D. catenulata* had light grayish-blue to pale purple-colored smooth colonies, and *D. cutanea* had dark blue colored smooth colonies. Additionally, none of these *Diutina* species produces a halo on CHROMagar Candida Plus (Fig. 2).

Antifungal susceptibility testing resulted in the following minimum inhibitory concentrations (MICs): amphotericin *B* < 0.12 µg/ml, 5-fluorocytosine < 0.06 µg/ml, fluconazole 4 µg/ml, itraconazole < 0.015 µg/ml, posaconazole 0.25 µg/ml, voriconazole 0.12 µg/ml, anidulafungin 0.12 µg/ml, caspofungin 0.25 µg/ml, and micafungin 0.06 µg/ml.

### Genomic and phylogenomic analyses

After quality control the obtained raw FASTQ sequence reads had a total read length of 480 298 423 bp with an N50/N90 of 7094/1734 bp. The assembled nuclear genome had a length of 11 035 057 bp and a coverage of 35X, and consisted of six contigs of 2 663 814, 2 462 312, 1 922 956, 1 409 496, 1 338 180, and 1 238 299 bp. The mitochondrial genome was found to be 26 144 bp in size and had a coverage of 2496X. The total genome size was 11 061 201 bp. The quality of the genome was assessed with compleasm and showed that it had a high completeness with 95.69% (2045/2137) single BUSCOs found, followed by 0.66% (14/2137) fragmented and 3.65% (78/2137) missing BUSCOs with the saccharomycetes database. For the higher rank Ascomycota database, a lower completeness was found, as 88.51% (1510/1706) of the BUSCOs was found in the genome, 1.93% (33/1 706) were fragmented, and 9.55% (163/1 706) were missing BUSCOs. Annotation of the *D. cutanea* genome with Helixer resulted in 5582 predicted genes.

**Figure 3.**
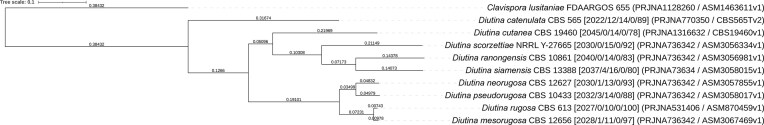
Phylogenomic analysis of the genus *Diutina* to identify the position of *Diutina cutanea* CBS 19460^T^. The genome of *D. cutanea* CBS 19460^T^ was generated within this study; all other reference genomes were obtained from the NCBI Genome database (https://www.ncbi.nlm.nih.gov/datasets/genome/). Numbers after the species name refer to the culture collection accession number; between square brackets the results of the compleasm analysis with the BUSCO saccharomycetes database showing the number of single, duplicated, fragmented class 1, fragmented class 2, and missing BUSCOs, respectively; and between parentheses the NCBI BioProject number and used assembly version. The branch lengths should be interpreted as the average number of substitutions per sites across all inferred gene trees.^18^

Phylogenomic analysis of the ten genomes resulted in 3502 orthogroups that contain genes of all included genomes, of which 2905 orthogroups contain a single copy per genome. The phylogenetic tree depicts the placement of *D. cutanea* CBS 19460^T^ within the genus *Diutina* (Fig. 3), being most closely related to *D. scorzettiae, D. ranongensis*, and *D. siamensis*. Moreover, we observed that the genomes of *D. rugosa* CBS 613^T^ and *D. mesorugosa* CBS 12656^T^ could not be phylogenetically resolved from each other (Fig. 3). Separate analysis of both genomes by OrthoFinder showed that out of the 5785 orthogroups, 5267 contain the same number of genes for *D. rugosa* and *D. mesorugosa*, further underlining their very closely relatedness.

Helixer gene prediction for all nine *Diutina* genomes resulted in an average of 5858 genes (±318). The smallest number of genes was found in *D. ranongensis* CBS 10861^T^ (*n*_genes_ = 5554), closely followed by the novel species *D. cutanea* CBS 19460^T^ (*n*_genes_ = 5582), *D. siamensis* CBS 13388^T^ (*n*_genes_ = 5581), *D. scorzettiae* NRRL Y-27665 (*n*_genes_ = 5665), *D. neorugosa* CBS 12627^T^ (*n*_genes_ = 5831), *D. mesorugosa* CBS 12656^T^ (*n*_genes_ = 5838), *D. rugosa* CBS 613^T^ (*n*_genes_ = 6019), *D. pseudorugosa* CBS 10433^T^ (*n*_genes_ = 6155), and the largest number by *D. catenulata* CBS 565^T^ (*n*_genes_ = 6501).

**Figure 4.**
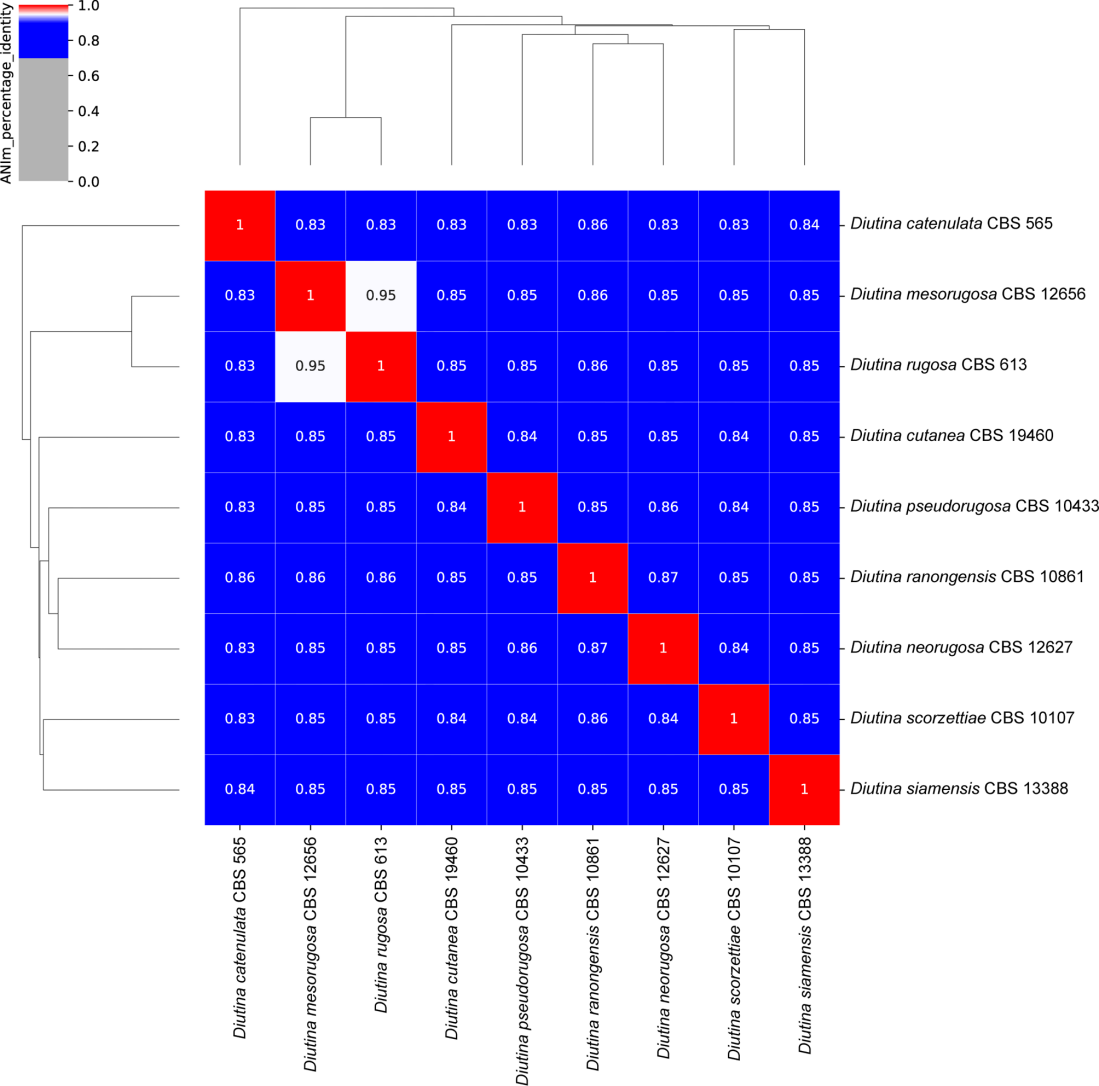
Average nucleotide identity analysis of the genus *Diutina* to identify the position of *Diutina cutanea* CBS 19460^T^. Genome assemblies used for phylogenomic analysis (see Fig. 3) were further investigated using pyani^19^ to obtain average nucleotide identity values for all combinations of genomes. There is at present not an established universal cutoff ANI-value for fungal genomes to mark species delineation.^25^

The ANI analysis shows that *D. catenulata* CBS 565^T^ is the least related to all other included *Diutina* genomes (Fig. 4). The genome of *D. cutanea* CBS 19460^T^ has an ANI-value of 0.84–0.85 versus the other eight *Diutina* genomes, which mostly have similar ANI-values. Furthermore, the ANI analysis supports the phylogenomic-based observation that *D. rugosa* CBS 613^T^ and *D. mesorugosa* CBS 12656^T^ have very similar genomes as the ANI-values were found to be 0.95 (Fig. 4).

An MSP was created to reliably identify *D. cutanea* from other *Diutina* species by using an in-house MALDI-TOF MS reference spectrum database. The MSP file has been made publicly available via various platforms (see Data availability section).

## Taxonomy

Description of *Diutina cutanea*, A. Zandijk, M. Groenew., and F. Hagen, sp. nov. (Fig. 1). MycoBank MB 861568. Etymology: Latin, *cutanea*, referring to skin, the clinical source from which the new species was first obtained.

After 7 days of growth on GYPA at 25°C, colonies were white, glossy, smooth, butyrous, and had an entire margin. The yeast cells were round to oval (2–3 × 3–8 µm) and occurred singly, in pairs, or clusters. Multilateral budding was observed. Slide cultures showed pseudohyphae formation at 25°C on GYPA after 7 days of incubation (Fig. 1). No sexual spores were observed on sporulation media after 1 month at 25°C. Carbon compounds assimilated are d-galactose, *N*-acetyl glucosamine, lactic acid, cellobiose, maltose, α,α-trehalose, potassium-2-keto-d-gluconate, mannitol, d-sorbitol, d-gluconate, d-glucose, and glucosamine (delayed). Carbon compounds not assimilated are sucrose, l-arabinose, raffinose, methyl-α-d-glucopyranoside, lactose, inositol, d-xylose, d-ribose, d-glycerol, l-rhamnose, erythritol, melibiose, d-glucuronate, melezitose, and l-sorbose. Nitrogen compounds assimilated are ethylamine, l-lysine, and cadaverine. Nitrogen compounds not assimilated are nitrite, nitrate, d-glucosamine HCl, and tryptophan. Fermentation of glucose, galactose, maltose, sucrose, lactose, raffinose, and trehalose is absent. Growth occurred at 6°C (weak) up to 37°C and is absent at 40°C. Growth was observed in the presence of 0.01% cycloheximide.

Holotype: CBS 19460 was isolated from a swab taken from a cutaneous wound of the fifth toe, May 2019, and preserved in a metabolically inactive state at the CBS Culture Collection hosted at the Westerdijk Fungal Biodiversity Institute, Utrecht, The Netherlands. Ex-type: B17938, 2MG-A1704-35.

## Discussion

We here described a novel species within the genus *Diutina* that we named *D. cutanea*, where the Latin adjective *cutanea*, refers to ‘of or pertaining to the skin’, as its source of isolation being a skin wound. Like some of the other *Diutina* species causing human infections (viz. *D. mesorugosa, D. neorugosa, D. pseudorugosa*, and *D. rugosa*), *D. cutanea* can grow at 37°C. However, *D. cutanea* could not grow at 40°C, while the aforementioned species can grow at up to 41°C.^3^

Antifungal susceptibility testing for the *D. cutanea* CBS 19460^T^ strain resulted in an MIC of < 0.12 µg/ml for amphotericin B, which is lower than reported for other *Diutina* species.^4,7–9,21,22^ The study by Ming et al.^4^ reported a 5-fluorocytosine MIC of 0.125 for all 24 *Diutina* isolates, while *D. cutanea* CBS 19460^T^ showed a decreased MIC for this antifungal compound of <0.06 µg/ml, which was also the reported MIC for *D. catenulata* by Chen et al.^4,8^ The *D. cutanea* CBS 19460^T^ strain had an MIC of <0.015 µg/ml for itraconazole, consistent with what has been previously reported.^4,7,8,21,22^ For voriconazole and posaconazole, the *D. cutanea* strain CBS 19460^T^ had an MIC of 0.12 and 0.25 µg/ml, respectively, which was comparable to previously MICs reported for *D. catenulata* and *D. rugosa*.^7–9,22^ For fluconazole, *D. cutanea* CBS 19460^T^ had an MIC of 4 µg/ml, the observed MICs for fluconazole were in the study by Ming et al.^4^ at least two 2-fold dilution steps lower (overall range 0.25–1 µg/ml).^4^ Stavrou et al.^22^ as well as Pérez-Hansen et al.^21^ reported an elevated MIC_50_ for *D. rugosa*, which was found to be 4 µg/ml.^21,22^ Elevated fluconazole MICs have recently been reported in various studies for *D. catenulata*, Nourrisson et al.^9^ reported an MIC_50_ of 3 µg/ml (range 0.75 to >256 µg/ml), Almeida-Paes et al. investigated a cluster of nine *D. catenulata* infections in a tertiary reference hospital and reported a fluconazole range of 4–32 µg/ml, while itraconazole (0.015–0.031 µg/ml), posaconazole (0.015–0.063 µg/ml), and voriconazole (0.063–0.25 µg/ml) had MIC ranges comparable to what was reported for *D. rugosa* (see above).^7,9^ For the echinocandins, *D. cutanea* CBS 19460^T^ is at the lower end of what has been reported for anidulafungin, caspofungin, and micafungin MIC_50_ and MIC_90_ values.^7–9,22^ Note that the above-referenced studies applied a variety of antifungal susceptibility testing methods (e.g., EUCAST or CLSI broth microdilution, or gradient strips), while the present study used the commercial SensiTitre YeastOne method. In a recent study by Ceballos-Garzon et al.,^23^ three antifungal susceptibility testing broth microdilution methods were compared to each other using 22 reference yeast strains and 13 antifungal compounds. This showed a high essential agreement between the CLSI, EUCAST, and SensiTitre YeastOne methods, except for fluconazole and 5-flucytosine that differed—after log2 transformation of the MIC-values—significantly between EUCAST and CLSI versus SensiTitre YeastOne. In these cases, the latter method yielded higher MIC values compared to EUCAST and CLSI.^23^ In the present study, the *D. cutanea* strain had comparable or lower MIC values compared to other *Diutina* species.

It has been reported that clinically relevant *Diutina* cannot be reliably discerned from each other using chromogenic media.^24^ However, the cited studies in that review were published well before a variety of chromogenic media came on the market to differentiate clinically relevant Saccharomycotina yeasts from each other. Here, we showed that CHROMagar Candida Plus can be used to differentiate clinically relevant *Diutina* species (Fig. 2).

Initially, sequencing of the ITS and D1/D2 regions of the ribosomal DNA identified that strain CBS 19460^T^ is a novel lineage within the genus *Diutina* as it had low identity scores of ≤89% with *Diutina* species present in NCBI GenBank. This is far below predicted taxonomic thresholds to discriminate yeast species, as this was found to be 98.41% and 99.51% for ITS and LSU, respectively.^25^ Hence, we applied long-read nanopore sequencing to obtain a *de novo* genome of *D. cutanea* CBS 19460^T^, subsequent phylogenomic analysis placed this proposed novel species distantly but basal to the non-pathogenic *Diutina* species *D. scorzettiae, D. ranongensis*, and *D. siamensis* (Fig. 3). The known pathogenic species form a separate group, here the position of *D. rugosa* CBS 613^T^ and *D. mesorugosa* CBS 12656^T^ stands out as they appear to be very closely related—with very short branch lengths—to each other compared to any of the other *Diutina* species (Fig. 3).

These phylogenomic observations were further substantiated by the ANI analysis, all species—except *D. rugosa* CBS 613^T^ and *D. neorugosa* CBS 12627^T^—showed ANI-values ranging between 0.83 and 0.86, while those for *D. rugosa* CBS 613^T^ and *D. mesorugosa* CBS 12656^T^ were found to be 0.95 (Fig. 4). Despite that ANI analysis is a well-established approach in bacterial taxonomy, it has not yet received similar appreciation within the field of fungal taxonomy.^26^ Recently, Cortimiglia et al.^27^ evaluated the use of ANI for various yeast genera and observed ANI-values of >0.92–0.98 for within species cutoff values and between-species cutoff values of <0.79 to <0.90%.^27^ Although the genus *Diutina* was not part of the study by Cortimiglia et al.,^27^ it can be derived from their between-species cutoff values that the observed ANI-values of the here analyzed *Diutina* genomes either identify them as individual species (*D. catenulata, D. cutanea, D. neorugosa, D. pseudorugosa, D. ranongensis, D. scorzettiae*, and *D. siamensis*) or likely as synonyms of one and the same species (*D. rugosa* and *D. mesorugosa*).^27^

Both the phylogenomic and ANI analyses support the previously reported observation that based on a multi-locus sequencing approach, a set of *D. mesorugosa* isolates could not be well separated from the *D. rugosa* isolates as the former fell between two clusters of the latter, and that both species were found to be minimally 98.4% identical to each other.^4^

Taken together, phylogenomic and ANI analyses showed that *D. cutanea* is a unique taxon with the genus *Diutina*. While, on the contrary, *D. rugosa* and *D. mesorugosa* are likely synonyms of the same genetic entity, but this needs to be further investigated. In a clinical setting, *D. cutanea* can be reliably discerned from other clinically relevant *Diutina* species by using CHROMagar Candida Plus and/or by using an enriched in-house MALDI-TOF MS database.

## Data Availability

Metadata related to strain CBS 19460^T^ (= 2MG-A1704-35 = B17938) is available via the database of the CBS Culture Collection hosted by the Westerdijk Fungal Biodiversity Institute, Utrecht, The Netherlands. The ITS and part of the 26S ribosomal DNA sequence data have been deposited in NCBI GenBank under accession number PX583673. The genome data have been deposited in NCBI under accession numbers PRJNA1316632 (BioProject), SAMN51115251 (BioSample), SRR35270397 (Sequence Read Archive), and JBQWSO000000000 (Genome). The Bruker MALDI BioType mass spectrum profile (MSP) file has been made available via the DANS-KNAW Data Station Life Sciences (https://doi.org/10.17026/LS/5PE63D), and will be included in the MicrobeNet database (https://microbenet.cdc.gov/).
